# Function of the *C. elegans* T-box factor TBX-2 depends on SUMOylation

**DOI:** 10.1007/s00018-013-1336-y

**Published:** 2013-04-18

**Authors:** Paul Huber, Tanya Crum, Lynn M. Clary, Tom Ronan, Adelaide V. Packard, Peter G. Okkema

**Affiliations:** 1 Department of Biological Sciences, Laboratory for Molecular Biology, University of Illinois at Chicago, Chicago, IL USA; 2 Department of Biology, Benedictine University, Lisle, IL USA; 3 Department of Biological Sciences, Harold Washington College, Chicago, IL USA; 4 Department of Biomedical Engineering, Washington University, St. Louis, MO USA

**Keywords:** T-box, SUMOylation, *C. elegans*, TBX-2, Genetic enhancer

## Abstract

T-box transcription factors are critical developmental regulators in all multi-cellular organisms, and altered T-box factor activity is associated with a variety of human congenital diseases and cancers. Despite the biological significance of T-box factors, their mechanism of action is not well understood. Here we examine whether SUMOylation affects the function of the *C. elegans* Tbx2 sub-family T-box factor TBX-2. We have previously shown that TBX-2 interacts with the E2 SUMO-conjugating enzyme UBC-9, and that loss of TBX-2 or UBC-9 produces identical defects in ABa-derived pharyngeal muscle development. We now show that TBX-2 is SUMOylated in mammalian cell assays, and that both UBC-9 interaction and SUMOylation depends on two SUMO consensus sites located in the T-box DNA binding domain and near the TBX-2 C-terminus, respectively. In co-transfection assays, a TBX-2:GAL4 fusion protein represses expression of a 5xGal4:tk:luciferase construct. However, this activity does not require SUMOylation, indicating SUMO is not generally required for TBX-2 repressor activity. In *C. elegans*, reducing SUMOylation enhances the phenotype of a temperature-sensitive *tbx*-*2* mutant and results in ectopic expression of a gene normally repressed by TBX-2, demonstrating that SUMOylation is important for TBX-2 function in vivo. Finally, we show mammalian orthologs of TBX-2, Tbx2, and Tbx3, can also be SUMOylated, suggesting SUMOylation may be a conserved mechanism controlling T-box factor activity.

**Electronic supplementary material:**

The online version of this article (doi:10.1007/s00018-013-1336-y) contains supplementary material, which is available to authorized users.

## Introduction

T-box proteins are a family of transcription factors found in all multicellular animals where they play important roles in the development of a variety of tissues [1, 2]. The defining feature of this family is the conserved T-box DNA-binding domain, and T-box factors are grouped into distinct sub-families based on sequence conservation within this domain. In many cases, the level of T-box factor activity is crucial to normal function. For example, reduced expression of the human Tbx2 sub-family genes *TBX3*, *TBX4,* and *TBX5* resulting from loss of one functional allele results in ulnar-mammary syndrome, small patella syndrome, and Holt-Oram syndrome, respectively [3–7]. In contrast, over-expression of the Tbx2-subfamily genes *TBX2* and *TBX3* is found in a number of human cancers [8]. Despite their developmental and clinical importance, relatively little is known about the mechanism by which T-box factors function.

We are interested in the role that SUMOylation plays in T-box factor activity. SUMOylation is the covalent and reversible post-translational attachment of the small ubiquitin-like modifier peptide (SUMO) to specific lysine residues in target proteins [9, 10], and it has been implicated in diverse processes, including modifying function, nuclear localization, and sub-nuclear localization of transcriptional regulators [11]. SUMOylation of transcription factors is typically associated with repression [12], but it has also been implicated in transcriptional activation by some factors [13, 14]. The SUMO conjugation pathway is analogous to the ubiquitination pathway and involves an E1-activating enzyme (Aos1/Uba2) and an E2 conjugating enzyme (Ubc9) sufficient for specific SUMO attachment in vitro [15, 16]. In addition, a variety of E3 ligases have been identified that promote SUMO transfer from E2 to specific substrates in vivo. Ubc9 recognizes the ΨKX(D/E) SUMO consensus site (where Ψ is a large hydrophobic amino acid and K is the residue attached to SUMO) [17, 18], and many SUMOylation substrates have been identified by their interaction with Ubc9 in yeast two-hybrid screens [19]. SUMOylation also occurs at non-consensus sites, and non-covalent SUMO/substrate or E3 ligase/substrate interactions are involved in directing SUMOylation at these sites [9].

We hypothesize that function of the *C. elegans* T-box factor TBX-2 depends on SUMOylation [20]. TBX-2 is the sole *C. elegans* member of the Tbx2 subfamily and is necessary for formation of anterior pharyngeal muscles. In yeast two-hybrid assays, TBX-2 interacts with the E2 SUMO conjugating enzyme UBC-9, and loss of UBC-9 produces pharyngeal phenotypes identical to those resulting from *tbx*-*2* loss-of-function. In addition, sub-nuclear localization of a TBX-2::GFP fusion protein is altered when SUMOylation is reduced.

Here, we ask if TBX-2 is SUMOylated and whether SUMOylation affects TBX-2 activity in vivo. We first used the two-hybrid assay to map interaction sites between TBX-2 and UBC-9 and found two SUMO consensus sites in TBX-2 that mediate interaction with UBC-9. One of these sites is located near the TBX-2 C-terminus, while the other is located in a highly conserved region of the T-box DNA binding domain. We next showed that TBX-2 is SUMOylated in mammalian cell assays, and that TBX-2 SUMOylation depends on both of these UBC-9 interaction sites. We then examined TBX-2 transcriptional activity and found that in mammalian cells a TBX-2-GAL4 DNA-binding domain (GAL4-DBD) fusion protein represses expression of a GAL4-responsive reporter, but surprisingly this repression did not require SUMOylation. To determine whether SUMOylation is important for TBX-2 activity in vivo, we asked if *tbx*-*2* and *ubc*-*9* interact genetically. We found that reduction of SUMOylation enhances the effect of a *tbx*-*2* hypomorphic mutant on embryonic viability and pharyngeal muscle development, and that repression of a downstream target of TBX-2 depends on SUMOylation. Finally, we examined SUMOylation of two mammalian orthologs of TBX-2 and found that human TBX2 and mouse Tbx3 can also be SUMOylated. We suggest SUMOylation is a common mechanism regulating activity of T-box transcription factors.

## Materials and methods

### Nematode handling, transformation, and strains

*C. elegans* were grown under standard conditions [21]. Germ line transformation was performed using standard techniques with pRF4 containing *rol*-*6(su1006)* as a dominant marker for transformation [22]. The following strains were used in these studies: OK0660 *tbx*-*2(bx59)* obtained by outcrossing from EM207 *tbx*-*2(bx59); him*-*5(e1490)*; OK0666 *cuEx553[D2096.6::gfp]*; OK0692 *tbx*-*2(bx59); cuEx553[D2096.6::gfp]*; OK0741 *tbx*-*2(ok529)/dpy*-*17(e164) unc*-*32(e189); cuEx553[D2096.6::gfp]*.

### Genotyping *tbx*-*2(bx59)* mutants

*tbx*-*2(bx59)* is a G→A substitution located at position 24,597 of the cosmid F21H11 (accession FO081200) (K. Chow, pers. comm.) and disrupts a BstCI restriction enzyme site. Animals were genotyped by single worm PCR [23] using primers PO931 [AGTTTGACACCGATTTTCTCG] and PO932 [GTGATGATGGATCTTGTTCCG] followed by digestion with BstCI and gel electrophoresis.

### General methods for nucleic acid manipulations and plasmid construction

Standard methods were used to manipulate plasmid DNAs and oligonucleotides [24], and all plasmid sequences are available from the authors. For yeast two-hybrid assays, the LKIE and VKKE SUMOylation sites were separately mutated using the Stratagene QuikChange II Kit in the *tbx*-*2* bait plasmid pOK187.01 containing the full-length *tbx*-*2* orf [20] to generate pOK222.01 and pOK222.06, respectively. The LKIE/VKKE→AAAA double mutant was constructed by ligation of fragments pOK222.01 and pOK226.06 to create the plasmid pOK225.02. Plasmids for expressing TBX-2 (pOK241.05), TBX-2^LKIE→AAAA^ (pOK241.10), TBX-2^VKKE→AAAA^ (pOK241.13), and TBX-2^LKIE/VKKE→AAAA^ (pOK241.17) were constructed by inserting the PCR amplified *tbx*-*2* orf from the two-hybrid vectors into pCDNA3.1 using TOPO cloning (Invitrogen). Plasmids for expressing TBX-2^K231R^ (pOK263.01), TBX-2^K400R^ (pOK244.18), and the TBX-2^2KR^ double mutant (pOK261.03) were made by site-directed mutagenesis of pOK241.05 using the Stratagene QuikChange II Kit. The plasmid encoding HA::SUMO-1 (pcDNA3 HA SUMO-1, pOK251.01) was a gift from Jorge A. Iñiguez-Lluhí (University of Michigan), and it was mutated using the Stratagene QuikChange II Kit to encode HA::SUMO-1(∆GG) (pOK263.05). cDNA clones for human TBX2 (IMAGE:6339405) and mouse Tbx3 (IMAGE:30547736) were purchased from Open Biosystems and inserted into pCDNA3.1 using TOPO cloning to make pOK246.01 and pOK245.01.

Plasmids for expressing TBX-2:GAL4 (pOK253.01) and TBX-2^LKIE/VKKE→AAAA^:GAL4 (pOK253.04) for co-transfection assays were made by cloning the amplified *tbx*-*2* orf from pOK241.05 and 241.17, respectively, into pcDNA HA:GAL4(1-100) (provided by Jorge A. Iñiguez-Lluhí, University of Michigan). For mock transfections, the HA:GAL4 fragment was removed from pcDNA HA:GAL4(1–100) (pOK293.03). The 5xGAL4:tk:luc reporter was a gift from Elizaveta Benevolenskaya, University of Illinois at Chicago.

### Yeast two-hybrid assays

Yeast 2-hybrid assays were carried out in L40 yeast containing HIS3 and *lacZ* reporters regulated by LexA binding sites with the *ubc*-*9* prey plasmid pOK193.11 in the pACT vector and *tbx*-*2* bait plasmids pOK187.01 (wild-type *tbx*-*2*), pOK222.01 (*tbx*-*2* ^LKIE→AAAA^), pOK222.06 (*tbx*-*2* ^VKKE→AAAA^), or pOK225.02 (*tbx*-*2* ^LKIE/VKKE→AAAA^) in the pLexA-NLS vector as previously described [20]. ß-galactosidase expression in yeast was quantified in at least three assays as previously described [25].

### RNAi analyses

Feeding RNAi was performed as previously described [26] using plasmids obtained from Geneservice containing genomic fragments of *ubc*-*9* or *smo*-*1* cloned into L4440 [27]. To assess enhancement of the *tbx*-*2(bx59)* mutant phenotype, N2 or OK0660 [*tbx*-*2(bx59)*] L4 hermaphrodites raised at 16 °C were transferred to plates seeded with RNAi feeding *E. coli* or OP50 and incubated at 25 °C for 24 h. These animals were transferred to fresh feeding plates at 25 °C and allowed to lay eggs for 4 h. Progeny embryos were transferred to fresh feeding plates and counted. Larvae and terminally arrested embryos were counted 24 h later to assay embryonic lethality, or examined by DIC microscopy after hatching to assess the pharyngeal phenotype.

To examine *D2096.6::gfp* expression, OK0666 [*cuEx553*] L4 hermaphrodites were transferred to plates seeded with *ubc*-*9* RNAi feeding *E. coli* or OP50 and grown 20 h at 20 °C. These animals were transferred to fresh feeding plates, and GFP expression was examined in progeny embryos and larvae.

### SUMOylation and co-transfection assays

COS-1 cells were maintained in D-MEM with 10 % FBS, 10 mM HEPES, and 1× Antibiotic–Antimycotic (Invitrogen). For SUMOylation assays, ~2 × 10^6^ cells were seeded into 10-cm plates 24 h prior to transfection. Plates were transfected with plasmids expressing wild-type or mutant TBX-2 (10 μg), HA-SUMO-1 or HA-SUMO-1(ΔGG) (10 μg), and peGFP-N3 (4 μg; Clontech) using Lipofectamine 2000 in OPTI-MEM following the manufacturer’s instructions (Invitrogen). After 48 h, COS-1 cells were harvested in PBS, lysed in 0.75 ml lysis buffer [8 M urea, 0.5 M NaCl, 45 mM Na_2_HPO_4_, 5 mM NaH_2_PO_4_,10 mM imidazole, 10 mM NEM (pH 8.0)], sonicated, and incubated with 50 μl Ni–NTA magnetic beads (Qiagen). Beads were washed twice with 1 ml wash [8 M urea, 0.4 M NaCl, 17.6 mM Na_2_HPO_4_, 32.4 mM NaH_2_PO_4_, 10 mM imidazole, 10 mM NEM (pH 6.75)] on a MagnaRack (Invitrogen). Protein was eluted in 50 μl (250 mM imidazole, 5 % SDS, 0.15 M Tris pH6.7, 30 % glycerol, 0.72 M ßME), resolved by SDS-PAGE, and blotted. Proteins were detected using anti-V5 (Invitrogen) or anti-HA (Covance), HRP-conjugated secondary antibody (Goat anti-mouse, Millipore), and ECL Plus (GE Healthcare) detection reagent. Chemiluminescence was recorded using ECL hyperfilm (GE Healthcare) or recorded and quantified using a STORM 860 Molecular Imager and ImageQuant software (Molecular Dynamics).

For co-transfection assays, 2 × 10^5^ COS-1 cells were seeded to wells of a 24-well plate 24 h prior to transfection. Wells were transfected with plasmids expressing TBX-2 (25–500 ng), 5xGAL4:tk:luc (280 ng), and CMV ßgal (20 ng) using Lipofectamine 2000 in OPTI-MEM following the manufacturer’s instructions (Invitrogen) and harvested. Luciferase and ß-gal were measured in triplicate samples using Steady Glo Luciferase system (Promega) and a Clarity Luminescence Micro-plate reader (BIO-TEK), and ßgal activity was measured with a Genesys 10 UV spectrophotometer (Thermo-Fisher) [28].

### Microarray and data analysis

Mixed stage populations of N2 and OK0660 [*tbx*-*2(bx59)*] animals grown at 25 °C were treated with bleach/sodium hypochlorite to isolate embryos [21]. Aliquots of embryos were examined to verify comparable age distributions, and RNA was isolated using TRIzol (Invitrogen) and further purified using RNeasy Kit (Qiagen) following manufacturers’ protocols.

Total RNA from two independent populations of N2 embryos and three independent *tbx*-*2(bx59)* embryos were labeled and hybridized to Affymetrix *C. elegans* Genome GeneChips by the UIC Core Genomic Facility (CGF). The microarray data was analyzed using the R statistical programming language, using the Bioconductor suite of tools [29], and the Affy package. Normalization to correct for chip-to-chip variation was done using the Robust Multiarray Averaging (RMA) method of microarray normalization [30]. Microarray results were pre-filtered using the genefilter function (25 % of the probes have a measured intensity of at least 100 on the original scale and the coefficient of variation is between 0.7 and 10 on the original scale) [31]. The limma package [32] was used to calculate differential expression using the limma linear model fit, eBayes smoothing of standard errors, and Benjamini-Hochberg (BH) multiple test correction with a false discovery rate of 5 % [33]. Probes were matched to genes using the Affymetrix-to-WormBase ID table for WS210 (http//:www.wormbase.org). Probes mapping to more than one gene were discarded. When one or more probes mapping to a gene were differentially expressed, that gene was considered to be differentially expressed. One GeneChip hybridized with *tbx*-*2(bx59)* RNA exhibited high variation in the spiked in control probes (TBXa) compared to the other samples, and data from this chip was not included in our analysis.

### Microscopy

Worms were visualized using a Zeiss Axioskop microscope equipped for DIC and fluorescence microscopy, and images were captured using an Axiocam MRm camera and AxioVision software.

## Results

### TBX-2 interacts with UBC-9 via two SUMO consensus sites

**Fig. 1:**
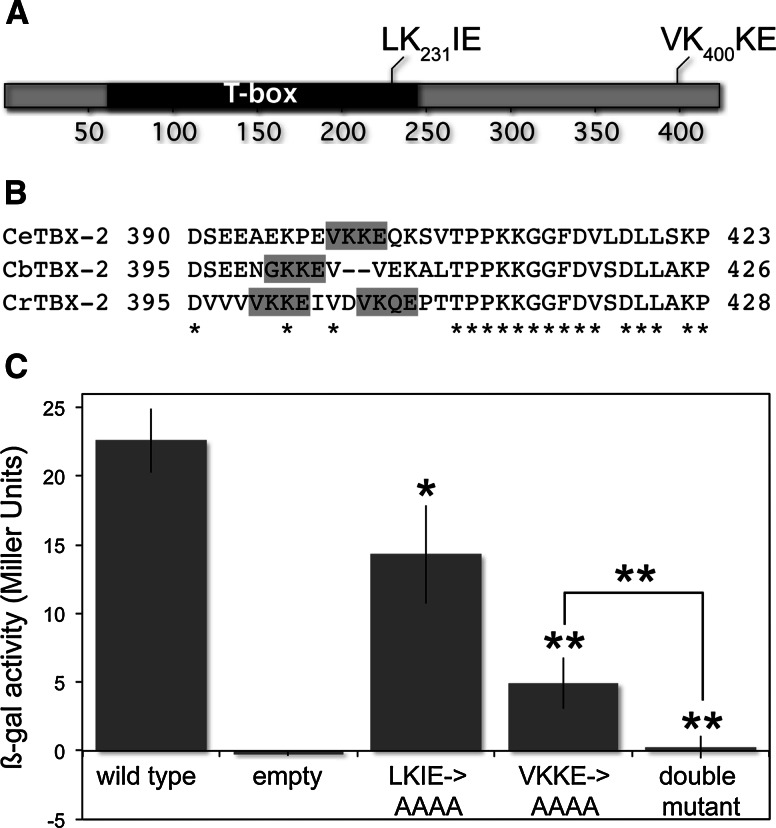
TBX-2 interacts with UBC-9. **a** Schematic diagram of the TBX-2 protein (Accession CCD69847) indicating the location of the T-box DNA binding domain (*black*) and the positions of the LK_231_IE and VK_400_KE SUMO consensus sites. **b** T-Coffee alignment of the C-terminus of TBX-2 proteins from *C. elegans* (CeTBX-2), *C. briggsae* (CbTBX-2; WormBase ID CBP05056), and *C. remanei* (CrTBX-2; WormBase ID RP21057) [62]. High-scoring SUMO consensus sites are indicated in *grey*, and identical residues are marked with* asterisks*. **c** Quantification of ß-galactosidase activity in yeast expressing the indicated TBX-2 protein or the empty pLexA as bait and UBC-9 prey in replicate samples from three independent experiments (*n* = 7). Differences between mutants and wild-type TBX-2 or different mutants (*bracket*) are statistically significant at *p < 0.05 or **p < 0.005. *Error bars* indicate the standard error of the mean

We previously showed using yeast two-hybrid assays that TBX-2 specifically interacts with the E2 SUMO-conjugating enzyme UBC-9 [20], and we used this assay to identify sites in TBX-2 mediating this interaction. TBX-2 contains several sites matching the SUMO consensus site ΨKX(D/E) (Supplementary Table 1) [17, 18]. The two highest-scoring matches to this consensus are an LK_231_IE sequence located near the C-terminus of the T-box DNA binding domain and a VK_400_KE sequence located near the TBX-2 C-terminus (Fig. 1a). LKIE is located in a conserved region of the T-box, and a SUMO consensus site is found at this position in many T-box factors, including all members of the Tbx2 sub-family [34]. VKKE is located in a region that is not highly conserved among T-box factors, although high scoring SUMO consensus sites are found near the C-terminus of TBX-2 proteins from *C. elegans*, *C. briggsae*, and *C. remanei*, suggesting this site may be functionally conserved (Fig. 1b). We mutated each of these two sites in *C. elegans* TBX-2 to all alanines either in single mutants (LKIE→AAAA or VKKE→AAAA) or in a double mutant (LKIE/VKKE→AAAA) and tested whether these mutants affected the ability of a TBX-2 bait to interact with UBC-9 prey. Interactions were scored in plate assays for histidine prototrophy and ß-galactosidase (ß-gal) expression, and the level of interaction was quantified by measuring ß-gal activity.

We found that UBC-9 interaction with TBX-2 was affected by mutations affecting both the LKIE and VKKE sites. UBC-9 interacted with both the TBX-2^LKIE→AAAA^ and TBX-2^VKKE→AAAA^ single mutants in plate assays, but this interaction was reduced to 63 and 22 % of the levels observed for wild-type TBX-2, respectively (Fig. 1b). In comparison, UBC-9 failed to interact with the TBX-2^LKIE/VKKE→AAAA^ double mutant in plate assays, and the ß-gal activity was close to that obtained using an empty bait plasmid (Fig. 1b, Supplementary Figure 1). As a control, we found that wild-type and all of the mutant TBX-2 proteins retained the ability to interact with an unrelated protein UNC-37 in yeast two-hybrid assays, indicating the mutant proteins were expressed (Supplementary Figure 1). Thus, both the LKIE and VKKE sites can interact with UBC-9. Because mutating both of these sites reduces interaction to near background, we believe they are the primary sites in TBX-2 that mediate this interaction. We have not tested other potential TBX-2 SUMOylation sites for interaction with UBC-9 in two-hybrid assays.

### TBX-2 can be SUMOylated in mammalian cell assays

To determine if TBX-2 can be SUMOylated, we co-expressed full-length TBX-2 and human SUMO-1 in COS-1 cells. TBX-2 was fused to poly-histidine and pulled down using Ni^2+^-beads under denaturing conditions, while TBX-2 and SUMO-1 were tagged with V5 and HA epitope tags, respectively, for detection on Western blots. A SUMO-1∆GG mutant lacking the C-terminal Gly–Gly motif required for conjugation to target lysine residues was used as a control to demonstrate SUMO conjugation.

**Fig. 2:**
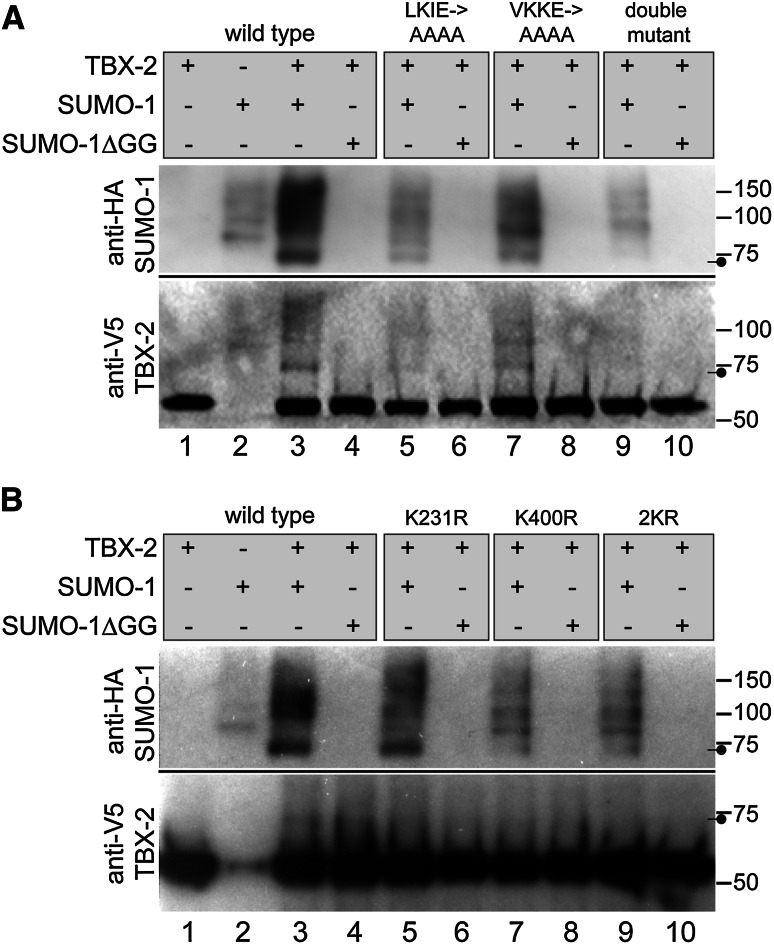
SUMOylation of TBX-2 is mediated via two SUMO consensus sites. Western blots of Ni–NTA pulled-down wild-type and mutant TBX-2/V5/HIS probed to detect TBX-2 (*bottom*) or SUMO-1 (*top*). Combinations of proteins (*grey boxes*) were expressed in COS-1 cells. **a** Wild-type TBX-2 and mutants with SUMOylation sites converted to all alanines. **b** Wild-type TBX-2 and mutants with SUMO acceptor lysines converted to arginines. The position of the fastest migrating SUMOylated form of TBX-2 is indicated (*bar and circle*) and the position of molecular weight markers are indicated in kDa (*bars*). Signal in the *lower panel* in A was detected using a STORM Molecular Imager and clearly demonstrates more slowly migrating TBX-2 bands when co-transfected with SUMO-1. TBX-2/V5/HIS is ~52 kDa, and HA-SUMO-1 is ~13 kDa. The fastest migrating SUMOylated form migrates somewhat slower than predicted by its molecular weight, which is a common feature of SUMOylated proteins

When co-expressed with SUMO-1, wild-type TBX-2 formed several more slowly migrating bands detectable with both anti-HA and anti-V5 (Fig. 2a). These bands likely represent mono- and multi-SUMOylated TBX-2, although proteins SUMOylated at different sites can also migrate at different positions due to the branched nature of the SUMOylated protein [35]. In comparison, no SUMOylated TBX-2 was detected when co-expressed with SUMO-1∆GG. As for many SUMOylated proteins, we found only a fraction of TBX-2 (~10 %) is SUMOylated in these assays. Similar results were obtained when TBX-2 was co-expressed with human SUMO-2 or SUMO-3 (Supplementary Figure 2).

We next asked how mutations in the UBC-9 interaction sites affected TBX-2 SUMOylation. SUMOylation of TBX-2^LKIE→AAAA^ and TBX-2^VKKE→AAAA^ were reduced to approximately 30 and 70 % of the level of wild-type TBX-2, respectively, while SUMOylation of the TBX-2^LKIE/VKKE→AAAA^ double mutant was further reduced to a level comparable to background (Fig. 2a, lanes 2 and 9). These results indicate that TBX-2 can be SUMOylated, and that the LKIE and VKKE sites for UBC-9 interaction are required for TBX-2 SUMOylation.

We next mutated the SUMO-conjugated lysine residues in the LKIE and VKKE sites to arginine, which is a conservative substitution that cannot be conjugated to SUMO, and we examined SUMOylation in COS-1 cells (Fig. 2b). The TBX-2^K231R^ mutant affecting LKIE exhibited reduction of the more slowly migrating SUMOylated products, but these products were not eliminated, while the fastest migrating band appeared unaffected. In comparison, overall SUMOylation of the TBX-2^K400R^ mutant affecting VKKE was strongly reduced, and the fast migrating form of SUMOylated TBX-2 was nearly completely eliminated. SUMOylation of the TBX-2^2KR^ double mutant containing K231R and K400R was similar to that of the TBX-2^K400R^ single mutant, but this mutant was still SUMOylated above background levels (compare Fig. 2b, lanes 2 and 9). Because both of these conservative mutations affect the pattern of TBX-2 SUMOylation, we believe lysine residues in both the LKIE and VKKE SUMO consensus sites are SUMOylated.

### TBX-2 is a transcriptional repressor in mammalian cells

**Fig. 3:**
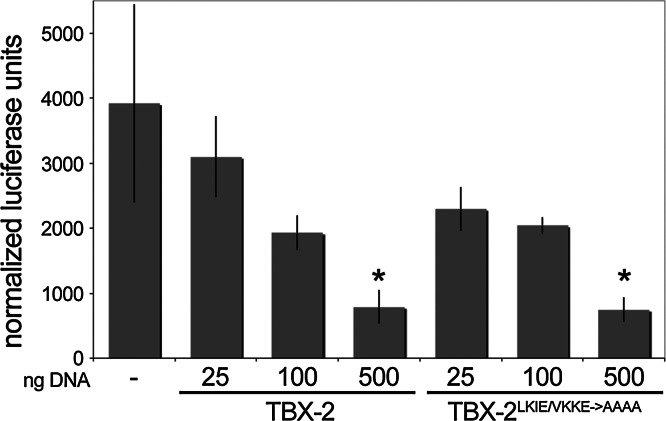
Dose-dependent transcriptional repression by TBX-2:GAL4. Relative luciferase activity in experiments co-transfecting increasing amounts of wild-type and mutant TBX-2:GAL4 with the 5xGAL4:tk:luc reporter. Data shown is the average of three assays and is representative of multiple independent experiments. *Error bars* indicate standard deviation. Statistically significant differences from control transfections are marked (*p < 0.05)

*C. elegans* TBX-2 is most closely related to the mammalian T-box repressors Tbx2 and Tbx3 [20]. We wanted to ask if TBX-2 functions similarly to repress transcription and, if so, whether this activity depends on SUMOylation. Because mutations affecting the LKIE SUMOylation would likely affect DNA binding, we asked if TBX-2 fused to the heterologous GAL4 DNA binding domain (TBX-2:GAL4) could repress expression of the 5xGAL4:tk:luc reporter. This reporter contains five copies of the GAL4 binding site upstream of thymidine kinase promoter:luciferase reporter, and TBX-2:GAL4 repressed expression of this reporter up to fivefold (Fig. 3). SUMOylation is most often associated with transcriptional repression, and we expected that mutation of the LKIE and VKKE SUMO sites would reduce this repressor activity. However, we found that the TBX-2^LKIE/VKKE→AAAA^ double mutant repressed 5xGAL4:tk:luc similarly to wild-type TBX-2. Co-expressing SUMO-1 did not affect repression of 5xGAL4:tk:luc with either wild-type or mutant TBX-2 (Supplementary Figure 3). Thus, SUMOylation is not required for TBX-2:GAL4 repressor activity in COS-1 cells.

### TBX-2 function is SUMO-dependent in *C. elegans*

**Fig. 4:**
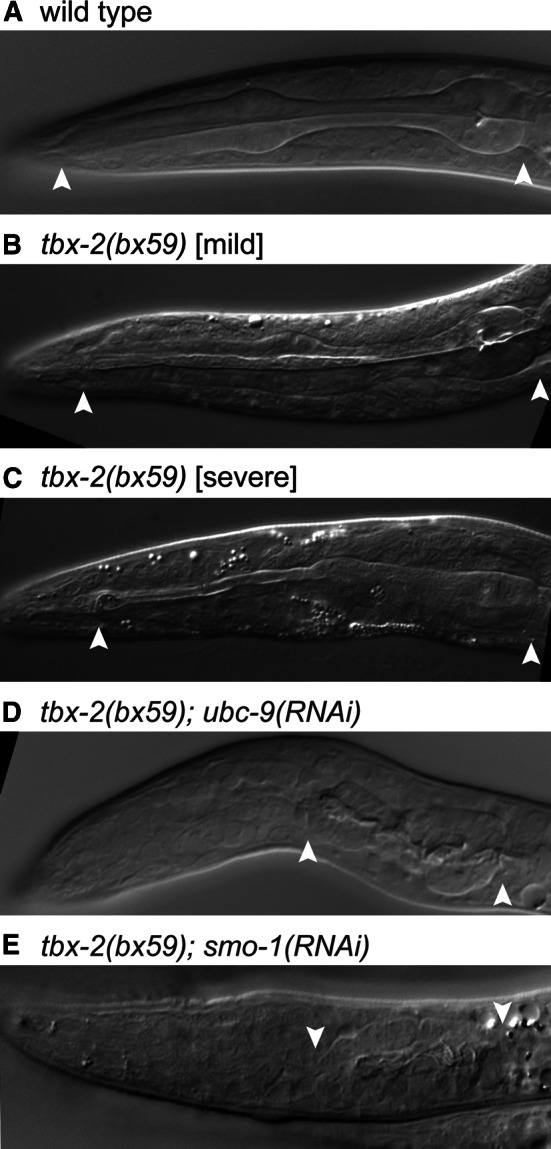
Pharyngeal defects in *tbx*-*2(bx59)* mutant are enhanced by reduced SUMOylation DIC micrographs of the pharynx of L1 larvae of the indicated genotypes raised at the non-permissive temperature (25 °C). **a** Wild-type N2. **b** *tbx*-*2(bx59)* exhibiting a mild pharyngeal defect. **c** *tbx*-*2(bx59)* exhibiting a more severe pharyngeal defect. *tbx*-*2(bx59); ubc*-*9(RNAi)* (**d**) and *tbx*-*2(bx59); smo*-*1(RNAi)* (**e**) L1 larvae exhibiting very severe defects resembling those of *tbx*-*2* null mutants. *Arrowheads* mark the extent of pharyngeal tissue. Anterior is left. The frequency of these phenotypes is indicated in Table 1

*mab*-*22(bx59)* is a temperature-sensitive mutant that exhibits defects in male tail ray formation and partially penetrant larval lethality. *bx59* has recently been identified as a missense mutation in *tbx*-*2* (King Chow, pers. comm.), and we subsequently refer to this mutation as *tbx*-*2(bx59)*. We examined the viability and pharyngeal morphology of *tbx*-*2(bx59)* mutants produced from hermaphrodites shifted to the non-permissive temperature (25 °C) at the L4 stage. Forty-four % of these animals arrested at the L1 stage (*n* = 64) with variable pharyngeal abnormalities (Fig. 4a–c). These phenotypes are similar to those observed in *tbx*-*2(RNAi)* animals and are not as severe as those observed in *tbx*-*2* null mutants [20, 36], and we conclude that *tbx*-*2(bx59)* is a hypomorphic allele.

We hypothesize that TBX-2 function depends on SUMOylation. To test this hypothesis, we asked if inhibiting SUMOylation by reducing UBC-9 or the SUMO protein SMO-1 by RNAi could enhance the phenotype of *tbx*-*2(bx59)* mutants. Using the RNAi-feeding method, we found that *ubc*-*9(RNAi)* produced a relatively low frequency of embryonic arrest in a wild-type background (Table 1) [26]. In comparison, *tbx*-*2(bx59); ubc*-*9(RNAi)* double mutants exhibited a synergistic increase in the frequency of arrested embryos (Table 1). *tbx*-*2(bx59); smo*-*1(RNAi)* double mutants also exhibited an increased frequency of embryonic lethality compared to each single mutant; however, the *smo*-*1(RNAi)* lethality alone was higher making it difficult to determine if the double mutant lethality was more than additive (Table 1).

**Table 1:** Reduction of SUMOylation enhances *tbx*-*2(bx59)* embryonic lethality and pharyngeal defects

Genotype^a^	% embryonic arrest (*n*)	Pharyngeal phenotypes in hatched L1s (percentage of total hatched animals)			
		Severe Tbx-2 pharynx	Mild Tbx-2 pharynx	Wild-type pharynx	*n*
*tbx*-*2(bx59)*	7 (120)	19	56	25	54
*ubc*-*9(RNAi)*	19 (186)	7	6	87	69
*tbx*-*2(bx59); ubc*-*9(RNAi)*	78 (200)	70	30	0	47
*smo*-*1(RNAi)*	65 (347)	22	39	39	49
*tbx*-*2(bx59); smo*-*1(RNAi)*	75 (359)	69	26	5	64

^a^L4 animals raised at 16 °C were shifted to 25 °C, and defects were scored in the F1 progeny

Many of the *tbx*-*2(bx59)* mutants that hatch grow to adulthood, but nearly all of the *tbx*-*2(bx59); ubc*-*9(RNAi)* and *tbx*-*2(bx59); smo*-*1(RNAi)* arrested as L1 larvae. We examined newly hatched larvae to determine if this enhanced L1 arrest results from pharyngeal defects. We found that both *tbx*-*2(bx59); ubc*-*9(RNAi)* and *tbx*-*2(bx59); smo*-*1(RNAi)* double mutants exhibited a synergistic increase in the frequency of animals with a severe anterior pharyngeal defect compared to the single mutants (Table 1; Fig. 4). Together, these results strongly suggest that SUMOylation is necessary for TBX-2 function for anterior pharyngeal development.

### TBX-2 and SUMOylation are required for repression of *D2096.6* gene expression

To identify genes downstream of TBX-2, we used microarrays to compare mRNA levels in populations of wild-type and *tbx*-*2(bx59)* embryos grown at 25 °C. We found 1,276 protein coding genes that are differentially expressed in *tbx*-*2(bx59)* (BH corrected *p* ≤ 0.05) (Supplementary Table 2). A total of 1,030 of these genes (80.7 %) are upregulated in *tbx*c-*2(bx59)*, consistent with our hypothesis that TBX-2 functions as a transcriptional repressor.

We focused on the gene *D2096.6*, which had previously been shown to be specifically expressed in the pharyngeal muscles, marginal cells, and epithelial cells under control of the FoxA-family transcription factor PHA-4 [37, 38]. We observed an approximately 1.8-fold increase in *D2096.6* expression in *tbx*-*2(bx59)* mutants in our microarray (BH corrected *p* = 0.03). While several candidate T-box binding sites are located upstream of *D2096.6*, our preliminary characterization of this promoter suggests it is indirectly regulated by TBX-2.

**Fig. 5:**
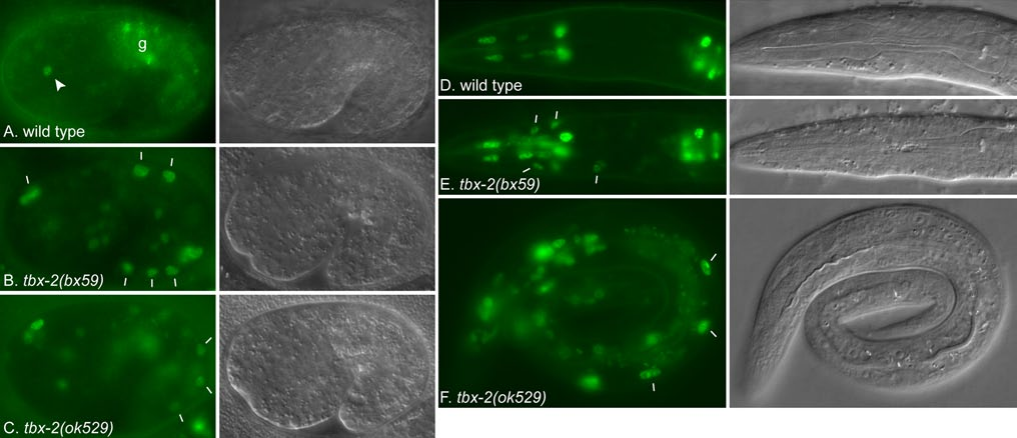
*D2096.6::gfp* is ectopically expressed in *tbx*-*2* mutants. Fluorescence (*left*) and DIC (*right*) micrographs of 1.5-fold stage embryos (**a**–**c**) and L1 larvae (**d**–**f**) expressing *D2096.6::gfp*. **a** *tbx*-*2(*+*); cuEx553[D2096.6::gfp]* embryo containing a single GFP-expressing pharyngeal nucleus (*arrowhead*) and auto-fluorescent gut granules (g). **b**, **c** *tbx*-*2(bx59); cuEx553* and *tbx*-*2(ok529); cuEx553* embryos exhibiting widespread *D2096.6::gfp* expression outside the pharynx. **d** *tbx*-*2(*+*); cuEx553* L1 with GFP expression in pharyngeal nuclei. **e**, **f** *tbx*-*2(bx59); cuEx553* and *tbx*-*2(ok529); cuEx553* L1 larvae. Representative bodywall muscle and hypodermal nuclei ectopically expressing *D2096.6::gfp* are marked (*bars*)

To determine how TBX-2 regulates *D2096.6* expression, we compared expression of a *D2096.6::gfp* reporter in wild-type and *tbx*-*2* mutants. Consistent with previous studies [37], we observed that a *D2096.6::gfp* reporter was expressed in wild-type embryos specifically in the pharynx at beginning approximately at the bean stage when the pharyngeal primordium forms. Expression was typically observed in one to two cells in the pharynx in one and one-half fold embryos (Fig. 5), and no expression was observed outside the pharynx. The number of GFP-expressing cells increased and animals hatched as L1s with GFP expression in pharyngeal muscles, marginal cells, and epithelial cells (Fig. 5d). In comparison, in *tbx*-*2(bx59)* and *tbx*-*2(ok529)* embryos *D2096.6::gfp* was expressed in more cells in the pharynx, and expression was observed in many cells outside the pharynx, including body wall muscles and hypodermal cells (Fig. 5b, c; Table 2). Ectopic *D2096.6::gfp* expression continued into the L1 larval stage where it was observed in bodywall muscle, hypodermal, and gut cells (Fig. 5e, f). These results indicate TBX-2 is an upstream regulator that represses *D2096.6* expression both temporally and spatially.

**Table 2:** Ectopic expression of *D2096.6::gfp*

Genotype	% animals with ectopic *D2096.6::gfp* expression (*n*)
*cuEx553[D2096.6::gfp]*	5 (136)
*tbx*-*2(bx59); cuEx553* ^a^	71 (55)
*tbx*-*2(ok529); cuEx553* ^b^	20 (35)
*ubc*-*9(RNAi); cuEx553*	75 (48)

^a^L4 animals raised at 16 °C were shifted to 25 °C, and defects were scored in the F1 progeny

^b^Progeny segregating from *tbx*-*2(ok529)/dpy*-*17(e164) unc*-*32(e189)* hermaphrodites were scored. Twenty-five % of these progeny are expected to be *tbx*-*2(ok529)* homozygotes

**Fig. 6:**
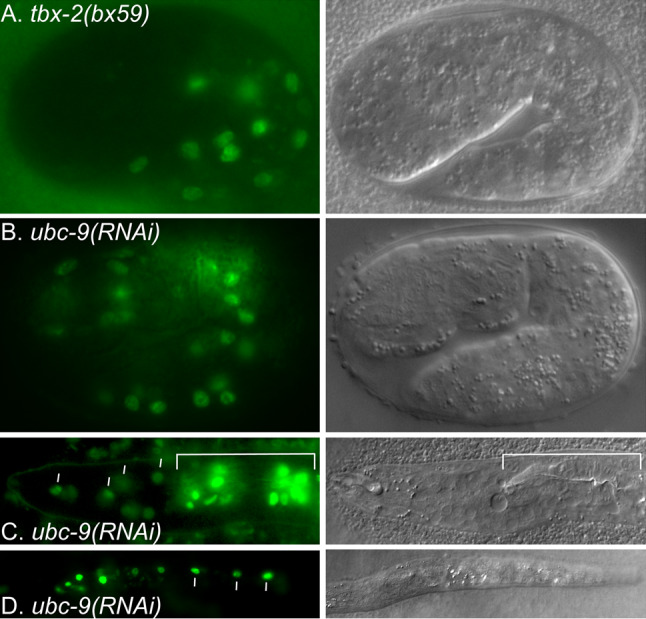
*D2096.6::gfp* is ectopically expressed in *ubc*-*9(RNAi)* animals. Fluorescence (*left*) and DIC (*right*) micrographs of a *tbx*-*2(bx59); cuEx553* embryo (**a**) and a *ubc*-*9(RNAi); cuEx553* embryo (**b**), and L1 larva (**c**, **d**) expressing *D2096.6::gfp* in bodywall muscle and hypodermal nuclei (*bars*). The *white bracket* indicates the partial pharynx in **c**

To ask if SUMOylation is necessary for TBX-2 function, we examined *D2096.6* expression in animals where activity of UBC-9 was reduced using feeding-RNAi. The most severely affected *ubc*-*9(RNAi)* animals have a highly disorganized morphology that makes it difficult to identify specific tissues [20]. Therefore we characterized *D2096.6::gfp* expression in older embryos that had undergone morphogenesis and the surviving L1 larvae. *ubc*-*9(RNAi)* resulted in *D2096.6::gfp* expression in posterior body wall muscles in embryos in a pattern similar to that which we have observed in *tbx*-*2(bx59)* and *tbx*-*2(ok529)* embryos (Fig. 6a, b). In larvae, we observed expression in body wall muscles and hypodermal cells in the posterior of the worm similar to the expression pattern we see in *tbx*-*2* mutants (Fig. 6 c, d). Thus, SUMO-dependent mechanisms repress *D2096.6::gfp* expression and the similarities in the pattern of ectopic expression in *ubc*-*9(RNAi)* and *tbx*-*2* mutants strongly suggests TBX-2 function depends on SUMOylation.

**Fig. 7:**
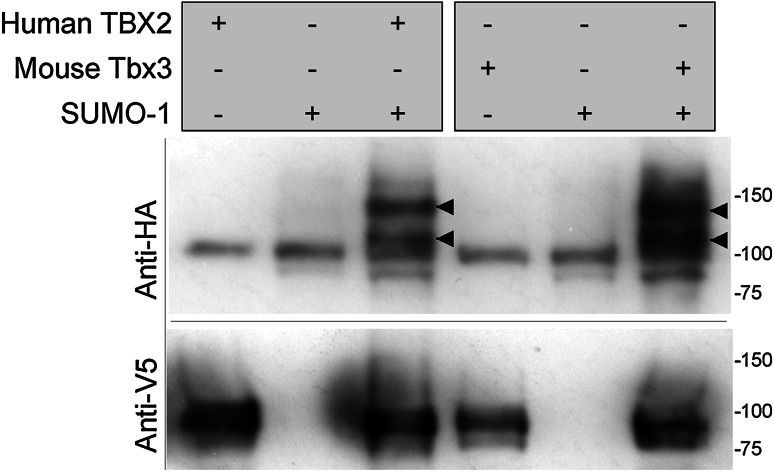
SUMOylation of mammalian Tbx2 subfamily members Western blots of Ni–NTA pulled-down human TBX2/V5/HIS and mouse Tbx3/V5/HIS probed to detect TBX2 and Tbx3 (*bottom*) or SUMO-1 (*top*). Combinations of proteins expressed in COS-1 cells are indicated (*grey boxes*). SUMOylated forms of TBX2 and Tbx3 are marked with *arrowheads*, and MW weight markers are indicated (kDa). A cross-reacting background band was detected in all lanes using anti-HA

### Mammalian Tbx2 subfamily members can be SUMOylated

To determine if SUMOylation is a conserved mechanism regulating T-box factor activity, we asked if mammalian orthologs of TBX-2 could be SUMOylated. Human TBX2 or mouse Tbx3 tagged with poly-histidine and V5 were co-expressed in COS-1 cells with or without HA:SUMO-1 and pulled down using Ni^2+^-beads under denaturing conditions similarly to *C. elegans* TBX-2. When co-expressed with SUMO-1, both TBX2 and Tbx3 formed two more slowly migrating SUMOylated bands (Fig. 7). This data indicates that other Tbx2-subfamily members can be SUMOylated, and we hypothesize that SUMOylation may be a common regulatory method of T-box factor activity.

## Discussion

Here we show that *C. elegans* TBX-2 and its mammalian orthologs human TBX2 and mouse Tbx3 can be SUMOylated, and that TBX-2 SUMOylation depends on two SUMO consensus sites that mediate interaction with the E2 SUMO conjugating enzyme UBC-9. We further demonstrate that *C. elegans* TBX-2 can function as a transcriptional repressor when fused to a heterologous DNA-binding domain; however, mutations that eliminate SUMOylation do not affect this repressor activity. Finally, we provide genetic evidence that SUMOylation is required for TBX-2 function in vivo by showing that reduction of SUMOylation enhances the phenotype of a hypomorphic *tbx*-*2* mutant and phenocopies the loss of *tbx*-*2* on expression of one gene that is downstream of TBX-2.

### Two TBX-2 SUMO consensus sites interact with UBC-9 and mediate SUMOylation

TBX-2 contains two predicted high-scoring SUMO consensus sites. VKKE located near the C-terminus and LKIE located within the T-box DNA-binding domain. Each of theses sequences interacts with the E2 SUMO-conjugating enzyme UBC-9 in yeast two-hybrid assays, and they are the only sites that can mediate this interaction.

Our data strongly suggests that both of these sites are SUMOylated. We observed multiple SUMOylated forms of TBX-2 in COS-1 cells, and mutations affecting either SUMO site reduce the amount of SUMOylated TBX-2. However, there are differences in how specific mutations in these sites affect TBX-2 SUMOylation. For VKKE, mutation of the acceptor lysine (K400R) results in a more severe reduction in SUMOylation than mutating this site completely to alanines. In comparison, mutating the LKIE site to alanines results in a large decrease in SUMOylation whereas mutation of the acceptor lysine (K231R) has a moderate affect and preferentially affects the more slowly migrating SUMOylated forms of TBX-2. Mutations affecting only the acceptor lysine likely retain interaction with UBC-9 (Sampson et al. 2001), whereas mutations converting the SUMO site to alanines eliminate UBC-9 binding. This difference likely underlies the different effects we observed on TBX-2 SUMOylation when these sites are mutated.

T-boxes have a highly conserved structure when bound to DNA [39–42], and TBX-2 LKIE is located within the α3 helix that spans the DNA backbone. While SUMOylation can occur in α helices [43, 44], this is an unusual 2˚ structure for UBC-9 interaction and SUMOylation, as UBC-9 has been shown to bind SUMO consensus sites in extended loops [18, 45]. However, recent evidence indicates that some T-boxes have significant structural flexibility that might allow SUMOylation at LKIE. The Tbx20 T-box exists as a molten globule with an unstable tertiary structure allowing flexibility between 2° structural domains [46]. Likewise in TBX5, the 3_10_-helix located just C terminal to the α3 helix is unstructured in the absence of DNA [42]. While the α3 helix remains structured when TBX5 is not bound to DNA, the LKIE site would be more accessible to bind UBC-9.

LKIE was also investigated as a potential SUMOylation site in the human T-box factor TBX22 [47]. Interestingly, mutation of this site eliminated TBX22 SUMOylation, however, this is believed to result indirectly from possible effects on DNA binding as was observed with several DNA-binding defective mutants. Biochemical analyses of SUMOylated T-box factors is necessary to explicitly determine if this site is SUMOylated in different proteins.

### *tbx*-*2* genetically interacts with *ubc*-*9* and *smo*-*1*

*tbx*-*2(bx59); ubc*-*9(RNAi)* and *tbx*-*2(bx59); smo*-*1(RNAi)* animals exhibit penetrant embryonic arrest and enhanced pharyngeal defects, indicating that reduced SUMOylation affects TBX-2 activity in vivo. In particular, the enhanced pharyngeal phenotype of these animals resembles that of *tbx*-*2* null mutants, strongly suggesting that *tbx*-*2* and SUMOylation function in the same pathway to specify pharyngeal muscle fate [48]. These observations are consistent with the hypothesis that TBX-2 function is SUMO-dependent.

In comparison, we do not know why enhanced embryonic lethality was observed in *tbx*-*2(bx59); ubc*-*9(RNAi)* and *tbx*-*2(bx59); smo*-*1(RNAi)* animals. Neither *tbx*-*2* null mutants nor *tbx*-*2(RNAi)* animals exhibit embryonic lethality [20], indicating lethality does not result from loss of zygotic *tbx*-*2(bx59)* activity. One possibility is that *tbx*-*2(bx59)* may have a partial gain-of-function character, and reducing SUMOylation deregulates this activity. *tbx*-*2(bx59)* mutates a conserved histidine residue within the dimerization domain of the T-box to a tyrosine (H145Y; accession CCD69847), and mutations affecting this domain in human TBX1 result in gain-of-function associated with some cases of DiGeorge and velocardiofacial syndromes [49]. Alternatively, decreased SUMOylation of a parentally provided, RNAi-resistant protein or mRNA *tbx*-*2* gene product might be required for viability. Finally, decreased SUMOylation may affect SUMO-dependent activity of both TBX-2 and another factor with a partially redundant activity required for embryogenesis.

### How might SUMOylation affect TBX-2 activity?

SUMOylation of transcription factors is usually associated with transcriptional repression, and it can promote recruitment of chromatin remodeling and histone modifying complexes [12, 50]. Indeed, SUMOylation of the *C. elegans* Ets-domain factor LIN-1 leads to interaction with MEP-1 and the NuRD chromatin repressor complex [51]. However, our results argue that SUMOylation affects TBX-2 function by a different mechanism. Unlike LIN-1, repressor activity of TBX-2:GAL4 fusion protein in mammalian cells is independent of SUMOylation, and in extensive yeast two-hybrid screens TBX-2 has not been observed to interact with MEP-1 [20].

SUMOylation at the LKIE site in the T-box would likely affect the ability of TBX-2 to bind sites in the genome and to interact with other factors binding TBX-2 regulated promoters. If this is the case, our assays for TBX-2:GAL4 repressor activity would be insensitive to SUMOylation, since they depend on a heterologous DNA-binding domain targeting a synthetic promoter. Indeed, SUMOylation has been shown to affect DNA-binding activity of specific transcription factors [52–54].

It is difficult to predict how SUMOylation at the VKKE site would affect TBX-2 activity. T-box proteins are poorly conserved outside of the DNA-binding domain, and the function of the TBX-2 C-terminus is unknown. Interestingly, our preliminary results indicate VKKE is located near an interaction site for the Groucho-family co-repressor UNC-37 (T. Crum and P. Okkema, unpublished). SUMOylation can regulate interaction with Groucho-family proteins [55, 56], and we hypothesize SUMOylation at VKKE has a similar function. Groucho-interaction motifs are enriched in T-box factors from *C. elegans*,* Drosophila*, and humans [57], and several T-box factors have been shown to interact with Groucho-family proteins [58–61]. SUMOylation may be a common mechanism for regulating T-box factor interaction with Groucho.

### Electronic supplementary material

Below is the link to the electronic supplementary material.

Supplementary Figure 1 (TIFF 3407 kb)

Supplementary Figure 2 (TIFF 295 kb)

Supplementary Figure 3 (TIFF 79 kb)

Supplementary Table 1 (DOCX 77 kb)

Supplementary Table 2 (XLSX 181 kb)
