# Supplementary figures and images for: Function of the C. elegans T-box factor TBX-2 depends on SUMOylation
Source: Cell Mol Life Sci. 2013 Apr 18;70(21):4157–68. doi: 10.1007/s00018-013-1336-y (PMC3802552; doi:10.1007/s00018-013-1336-y)

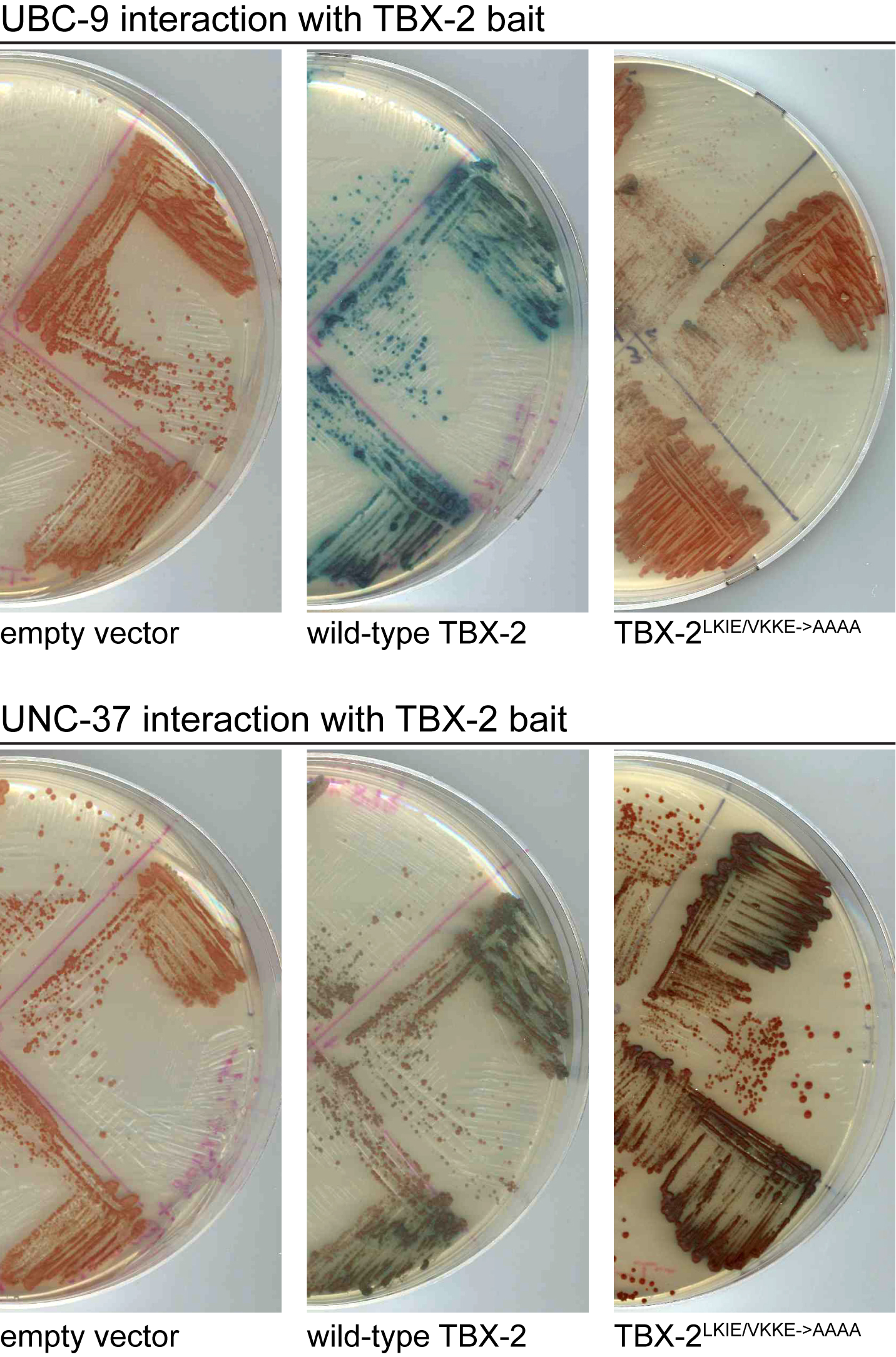

Supplement: Supplementary file 1 — Supplementary Figure 1 (TIFF 3407 kb) [file 18_2013_1336_MOESM1_ESM.tif]

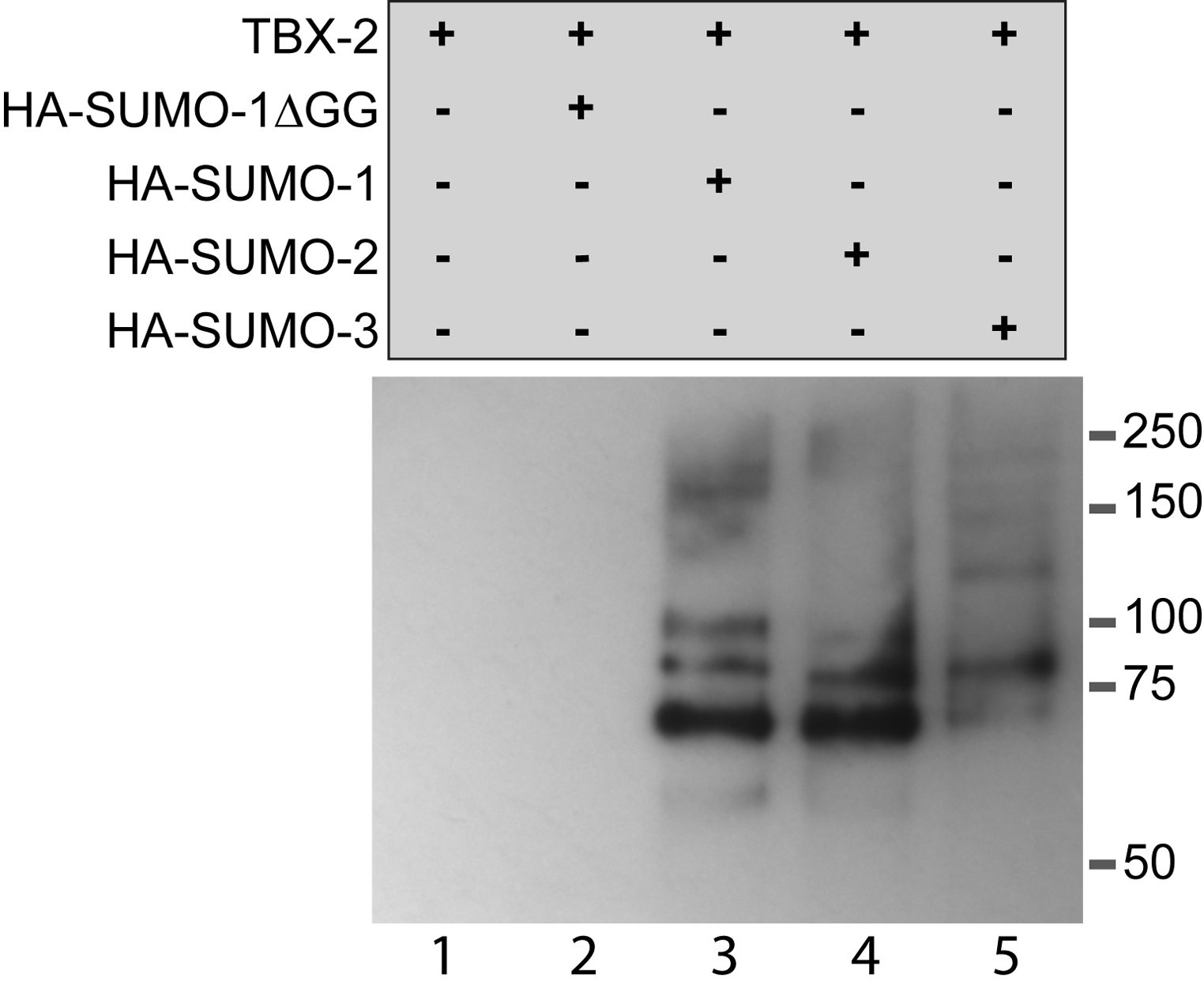

Supplement: Supplementary file 2 — Supplementary Figure 2 (TIFF 295 kb) [file 18_2013_1336_MOESM2_ESM.tif]

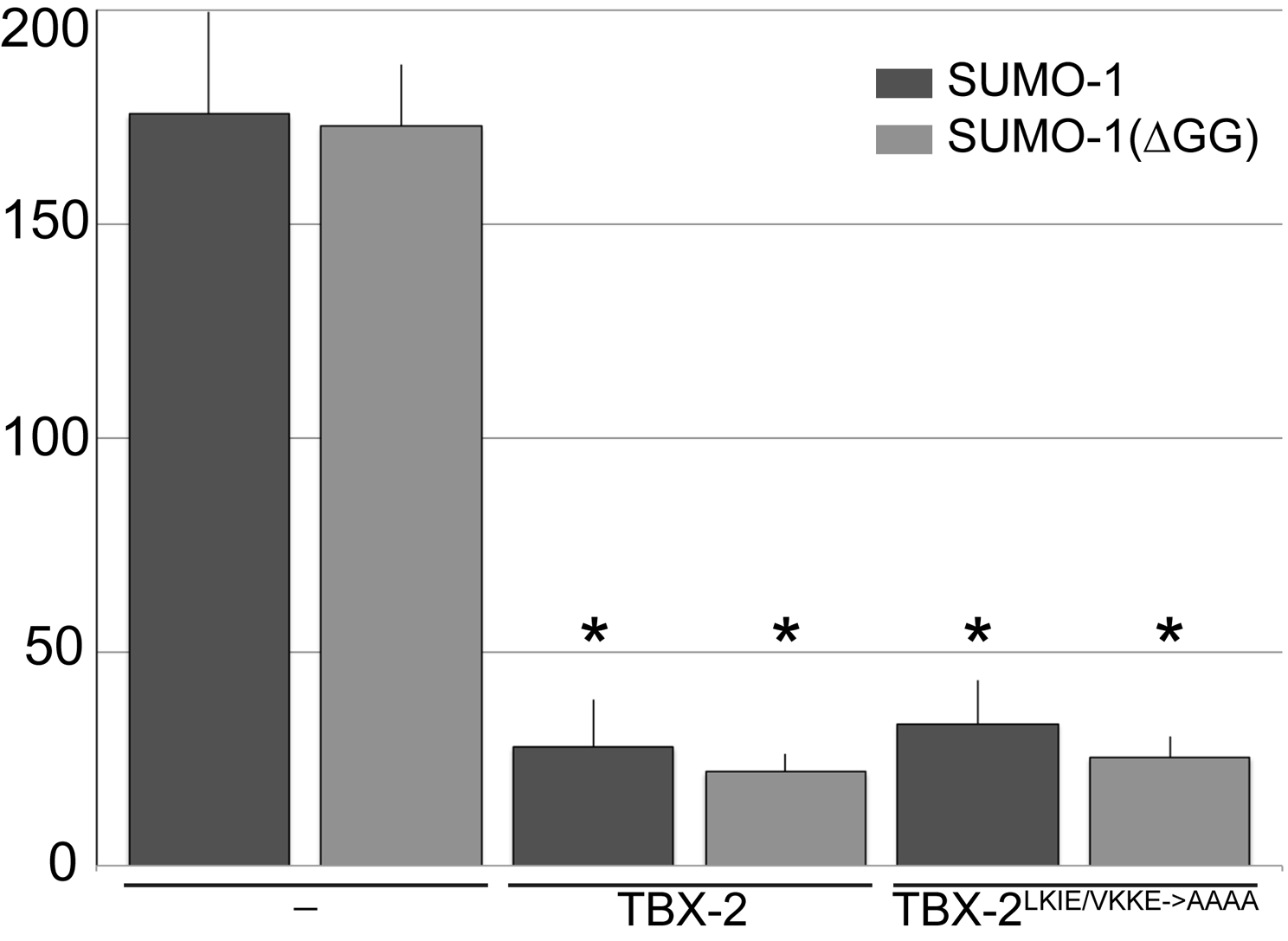

Supplement: Supplementary file 3 — Supplementary Figure 3 (TIFF 79 kb) [file 18_2013_1336_MOESM3_ESM.tif]
