# Supplementary material for: Function of the C. elegans T-box factor TBX-2 depends on SUMOylation
Source: Cell Mol Life Sci. 2013 Apr 18;70(21):4157–68. doi: 10.1007/s00018-013-1336-y (PMC3802552; doi:10.1007/s00018-013-1336-y)
Supplement: Supplementary file 4 — Supplementary Table 1 (DOCX 77 kb) [file 18_2013_1336_MOESM4_ESM.docx]

Supplementary Table 1: Potential SUMOylation sites predicted by SUMOplot, SUMOhydro and SUMOsp 2.0

| **Rank** | **Position** | **Group** | **SUMOplot Score** |
| --- | --- | --- | --- |
| 1 | K400 | AEKPE **VKKE** QKSVT | 0.93 ^a^ |
| 2 | K231 | EKVTE **LKIE** NNPFA | 0.91 ^a^ |
| 3 | K133 | RWMIA **GKAD** PEMPK | 0.67 |
| 4 | K248 | RDAGA **GKRE** KKRQL | 0.67 |
| 5 | K62 | GVTDD **PKVE** LDERE | 0.61 ^a^ |
| 6 | K396 | DSEEA **EKPE** VKKEQ | 0.50 |
| 7 | K239 | ENNPF **AKGF** RDAGA | 0.44 |
| 8 | K410 | KSVTP **PKKG** GFDVL | 0.43 |
| 9 | K174 | TNNIS **DKHG** YTILN | 0.33 |
| 10 | K411 | SVTPP **KKGG** FDVLD | 0.31 |

The TBX-2 protein sequence (Accession CCD69847) was analyzed for matches to the SUMO consensus site ΨKX(D/E) using SUMOplot at http://www.abgent.com/tools/. The top-scoring matches VKKE and LKIE were characterized in this study.

^a^ Also predicted as a potential SUMOylation site by SUMOhydro and SUMOsp 2.0 at high threshold [[63](#_ENREF_63),[64](#_ENREF_64)].
